# Chemical and Antimicrobial Analyses of *Sideritis romana* L. subsp. *purpurea* (Tal. ex Benth.) Heywood, an Endemic of the Western Balkan

**DOI:** 10.3390/molecules22091395

**Published:** 2017-08-23

**Authors:** Vanja Tadić, Alessandra Oliva, Mijat Božović, Alessia Cipolla, Massimiliano De Angelis, Vincenzo Vullo, Stefania Garzoli, Rino Ragno

**Affiliations:** 1 Institute of Medicinal Plants Research Dr Josif Pančić, Tadeuša Košćuška 1, 11000 Belgrade, Serbia; vtadic@mocbilja.rs; 2 Department of Public Health and Infectious Diseases, Sapienza University, P.le Aldo Moro 5, 00185 Rome, Italy; alessandra.oliva@uniroma1.it (A.O.); alessia.cipolla@hotmail.it (A.C.); massimiliano.deangelis@yahoo.com (M.D.A.); vincenzo.vullo@uniroma1.it (V.V.); 3 Faculty of Natural Sciences and Mathematics, University of Montenegro, Džordža Vašingtona bb, 81000 Podgorica, Montenegro; 4 Rome Center for Molecular Design, Sapienza University, P.le Aldo Moro 5, 00185 Rome, Italy; 5 Department of Drug Chemistry and Technology, Sapienza University, P.le Aldo Moro 5, 00185 Rome, Italy; stefania.garzoli@uniroma1.it

**Keywords:** *Sideritis romana* L. subsp. *purpurea* (Tal. ex Benth.) Heywood, essential oil, solvent extracts, GC-MS, flavonoids, phenols, tannins, antimicrobial activity

## Abstract

A comprehensive study on essential oil and different solvent extracts of *Sideritis romana* L. subsp. *purpurea* (Tal. ex Benth.) Heywood (Lamiaceae) from Montenegro is reported. The gas chromatography-mass spectrometry analysis of the essential oil revealed a total of 43 components with bicyclogermacrene (23.8%), germacrene D (8%), (*E*)-caryophyllene (7.9%) and spathulenol (5.5%) as the major ones. Sesquiterpenoid group was found to be the most dominant one (64.8%), with 19.9% of the oxygenated forms. In the crude methanol extract of the investigated plant, obtained by Sohhlet exraction, the total phenol content was 14.7 ± 0.4 mg of GA/g, the total flavonoids were 0.29 ± 0.03% expressed as hyperoside percentage, whereas the total tannins content was 0.22 ± 0.04% expressed as pyrogallol percentage. For the antimicrobial activity determination, the following microorganisms have been used: methicillin-susceptible *Staphylococcus aureus* (MSSA (American Type Culture Collection (ATCC) 29213)) and methicillin-resistant *S. aureus* (MRSA (clinical strain)), *Escherichia coli* (ATCC 25922), carbapenem-susceptible *Klebsiella pneumoniae* (clinical strain), carbapenem-resistant *K. pneumoniae* (clinical strain) and *Candida albicans* (ATCC 14053). The essential oil showed high potency against MSSA and MRSA, both at high (~5 × 10^5^ CFU/mL) and low (~5 × 10^3^ CFU/mL) inoculum. With respect to MSSA, the minimal inhibitory concentration (MIC) value was 0.307 mg/mL, with bactericidal activity obtained at 0.615 mg/mL, while, in the case of MRSA, the MIC and minimal bactericidal concentration (MBC) values were 0.076 and 0.153 mg/mL, respectively. Regarding anti-*Candida albicans* activity, the MIC value was 2.46 mg/mL without reaching fungicidal activity. In addition to the observed essential oil efficacy, different solvent extracts were analyzed for their antimicrobial activity. Similarly to the essential oil, thehighest efficacy was observed against both MSSA and MRSA strains, at high and low inoculums, in the case of the 1,2-dichloroethane and methanol extracts. A potent fungicidal activity has been also found for the *n*-hexane and 1,2-dichloroethane extracts. It can be concluded that *Sideritis romana* L. subsp. *purpurea* (Tal. ex Benth.) Heywood provides a wide range of application in different fields such as phytochemistry, pharmacology, toxicology or pharmacognosy.

## 1. Introduction

Essential oils (EOs) have been known since antiquity to possess different biological activities, notably antibacterial and antifungal, or antioxidant properties [1,2]. Generally, these activities depend on the chemical composition of EO that is determined by the plant genotype, but also greatly influenced by several factors such as geographical origin and environmental and agronomic conditions [3,4,5]. Many EOs and their ingredients have an unexpectedly large range of applications. Their cosmetic use is probably the most ancient one, both for their functional, perfume and preservative properties [6]. They have been widely used as food flavors [7], and possessing antioxidant and antimicrobial activities serve as natural additives in foods and food products [8]. Known for their antiseptic (i.e., bactericidal, virucidal and fungicidal), medicinal properties and their fragrance, EOs are used in embalmment, preservation of foods and as antimicrobial, analgesic, sedative, anti-inflammatory, spasmolytic and local anesthetic remedies [9]. 

*Sideritis romana* L. subsp. *purpurea* (Tal. ex Benth.) Heywood (SP) is an endemic of the Western Balkan. Although numerous scientific studies related to different *Sideritis* species confirmed biological effects of their active compounds [10,11,12,13,14,15,16,17,18,19,20], such studies considering this species are rather rare. To the best of our knowledge, there is only one report for SP collected in Greece [21]. Herein, we report for the first time a comprehensive study on its chemical composition and the related antimicrobial activity from material collected in Montenegro.

### Taxonomic Characterization and Uses of SP

**Figure 1:**
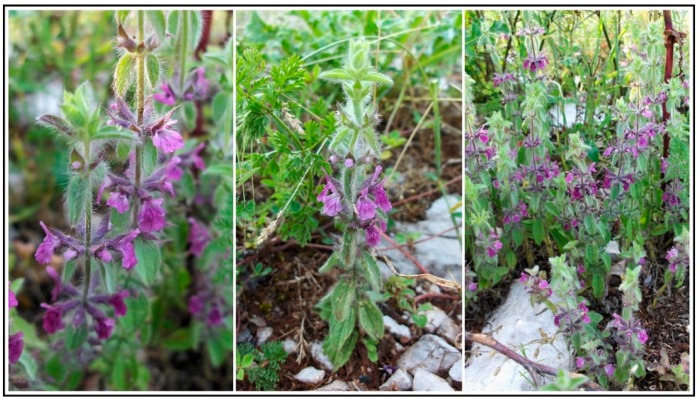
*Sideritis romana* L. subsp. *purpurea* (Tal. ex Benth.) Heywood in full blossom on its natural habitat in Šteke locality near Podgorica city (Montenegro) (photo by Mijat Božović).

Purple ironwort [synonyms: *S. purpurea* Tal. ex Benth., *Hesiodia purpurea* (Tal. ex Benth.) Sojàk] from Lamiaceae family is a densely villous-lanate annual that grows up to 30 cm. Its stem is eglandular, and leaves are 10–25 × 5–12 mm, oblong-ovate, dentate or crenate-dentate. Verticillasters are usually 6-flowered and distant. Calyx is 6–10 mm, 2-lipped; upper tooth is broadly ovate, lower lanceolate, all with a straight, pungent apex. Purple (or rarely white) corolla is 7–10 mm, equaling or slightly exceeding calyx, with the upper lip of 4–5 × 1–2 mm (Figure 1) [22].

It inhabits dry, rocky places or abandoned meadows and arid grasslands, but can also be found on the edges of irrigation ditches or alleys, usually at lower altitudes. To the best of our knowledge, there is no particular report on any traditional use of this subspecies. However, different biological activities of other *Sideritis* species have been investigated by numerous authors [10,11,12,13,14,15,16,17,18,19,20]. A review work [23] on genus *Sideritis* has reported that these plants have been traditionally used for tea preparation, as flavoring agents, and in folk medicine in the Mediterranean and Balkan regions as anti-inflammatory, antiulcerative, antimicrobial, anticonvulsant, antispasmodic, antioxidant, vulnerary, analgesic and carminative agents. According to the authors, diterpenes, flavonoids and EOs occurring in almost each species are the compounds mainly responsible for the observed in vivo and in vitro pharmacological activities. *Sideritis* is used in Turkish folk medicine as beverage in brew form for its high antibacterial, carminative, diuretic and digestive properties [24]. The species from this genus are used for its vulnerary properties to heal cuts and wounds of the skin [25], while, in some Italian regions, the decoy of the exsiccated leaves is used to wash the unconcealed parts of the body in case of fright in order to soothe anxiety [26].

## 2. Results and Discussion

### 2.1. EO and Solvent Extractions

SP essential oil (SPEO) isolated by hydrodistillation from the crushed dried plant material was obtained as a yellow liquid with a yield of 0.015% *w*/*w* (calculated per weight of dried material). 

Solvent extracts yields are shown in Table 1 and Table 2. In general, the methanol extracts furnished the highest yields.

### 2.2. EO Chemical Analysis

Detected components and their percentages according to their relative retention indices (RI) are given in Table 3. A total of 43 components was determined, representing 90.4% of the EO. The major components were bicyclogermacrene (23.8%), germacrene D (8%), (E or β, but one throughout the text)-caryophyllene (7.9%) and spathulenol (5.5%). Sesquiterpenoid group was found to be the most dominant one (64.8%), with 19.9% of the oxygenated forms. Mono- and diterpene fractions were present only with oxygenated forms: 4.4% and 5.7%, respectively.

Many studies have been performed on the chemical composition of EOs from different *Sideritis* species, explaining the polymorphism among the populations, as well as the existence of new species, chemical varieties and hybrids. Numerous studies have reported the influence of the geographical location, season, climatological variations, plant variety and experimental conditions on EO composition of *Sideritis* species, and a possible existence of chemotypes or ecotypes has also been noted [27,28,29,30]. The most common terpene group of constituents among *Sideritis* species is almost absent, since some species are rich in monoterpenoids, while others are characterized by the prevalence of sesquiterpenoid fraction, or even diterpenes [21,31]. According to the most prevalent compounds, some authors classified *Sideritis* species into six main groups: monoterpene hydrocarbon-rich, oxygenated monoterpene-rich, sesquiterpene hydrocarbon-rich, oxygenated sesquiterpene-rich, diterpene-rich and others [31].

High content of monoterpene hydrocarbons has been reported in many studies, with α-pinene, β-pinene, sabinene, myrcene or limonene as the main ones [27,30,31,32,33]. Thus, some Greek endemic *S. clandestina* subspecies are rich in α- and β-pinene [21], as well as *S. congesta* and *S. argyrea* EOs [34]. The Spanish endemic *S. ibanyezii* has been found to be rich in sabinene and α-pinene [32,35], and the same monoterpenes along with fenchone and cineole were in prevalence in *S. pusilla* from the Iberian Peninsula [36]. Limonene was found as the major compound in *S. perfoliata* [34], and the prevalence of myrcene has been reported for *S. syriaca* [37].

Oxygenated derivatives are not common as main constituents among *Sideritis* species. Oxygenated forms of monoterpenes are characteristic constituents only in several species: *S. arguta, S. libanotica* and *S. romana* [31,38]. Oxygenated sesquiterpenes predominate in EOs of *S. phlomoides* and *S. taurica* [31], while in the group rich in sesquiterpenes, β-caryophyllene, germacrene D and calamene have been identified as the main constituents [19]. Thus, the Greek endemic *S. euboea* was dominated by the presence of sesquiterpenes, with the oxygenated sesquiterpene valeranone being the main component, followed by β-caryophyllene and γ-muurolene [21]. Sesquiterpenoids β-caryophyllene and nerolidol dominated in *S. scardica* [37], while germacrene D and bicyclogermacrene were found in prevalence in *S. montana, S. curvidens* and *S. raeseri* [31,37,39]. *S. lanata* was also characterized by the abundance of sesquiterpenes, with spathulenol and β-phellandrene being the major metabolites [21,31], while *S. condensata* provided an EO with high proportions of β-caryophyllene and α-pinene [34]. *S. perfoliata* and *S. dichotoma* are rich in diterpenes [40], as well as an Greek endemic *S. clandestina* subsp. *peloponnesiaca* with the labdane derivative isoabienol being the major diterpene constituent [21]. *S. lanata* formed a distinct group with the occurrence of a fatty acid as main constituent in the EO [31].

To the best of our knowledge, there is only one report for SPEO from plant collected inGreece [21]. That analysis also showed predominance of sesquiterpene fraction (83.6%) with bicyclogermacrene (48.9%), β-caryophyllene (12.7%) and γ-muurolene (11.9%) as the major ones. Monoterpenes were present in 16.2% with β-pinene (7.9%) being the main metabolite. The results presented herein are partly in compliance with the ones previously reported. It is also worth noting that the other subspecies *romana* was found to be rich in oxygenated monoterpenes with thymol (24.9%) being the most abundant in the material from Turkey [31] and carvacrol (20%) in the Italian-origin material [38]. Montenegrin SP can thus be described as the sesquiterpenoids-rich one, with 10.1% of the oxygenated mono- and diterpene fractions being less important.

### 2.3. Total Phenolic, Flavonoid and Tannin Contents

Medicinal plants are known to produce diverse substances possessing antioxidant properties that have the ability to protect the human body against cellular oxidation [41]. Antioxidant compounds as different classes of secondary metabolites like polyphenolic acids, different groups of flavonoids, including flavonols, flavons, flavanones, flavanonols, isoflavonoids, antocyanidis, derivatives of flavan 3-ols (tannins), flavan-3,4-diols, are well known to be able to scavenge free radicals and thus inhibit the oxidative mechanisms that lead to degenerative diseases [42]. 

Phenolic compounds are a class of antioxidants that act as free radical terminators and their bioactivities may be related to their abilities to chelate metals, inhibit lipoxygenase and scavenge free radicals [43]. The phenol content of a plant depends on a number of intrinsic (genetic, extracting solvent) and extrinsic (environmental, handling and development stage) factors [44]. Total phenolic content (TPC) in the methanol SP extract (F) using the Folin–Ciocalteu’s reagent was expressed in terms of gallic acid equivalent (mg of GA/g of extract) and it was 14.7 ± 0.4. Similar results were obtained with *S. lycia* and *S. libanotica* being 16.05–18.04 and 9.16–10.49 mg GA/g, respectively [45], while higher TPC has been reported for *S. raeseri* (15.3 to 34.1 mg GA/g), *S. scardica* (188.5 ± 12.9 mg GA/g), *S. amasiaca*, *S. serratifolia* (from 402.5 ± 2.5 to 321.1 ± 0.5 mg GA/g), *S. condensata* and *S. eryhrantha* samples (from 247.62 ± 1.91 to 217.61 ± 0.95 mg GA/g) [20,39,46,47,48]. It is well-known that phenolic compounds contribute to quality and nutritional value in terms of modifying color, taste, aroma, and flavor and also in providing health beneficial effects. They also serve in plant defense mechanisms to counteract reactive oxygen species in order to survive and prevent molecular damage and damage by microorganisms, insects, and herbivores [49,50,51].

Although a group of compounds of relatively homogenous structure, the flavonoids exhibit wide range of activities, antimicrobial being one among others. Flavonoids were found to reduce blood-lipid and glucose and to enhance human immunity, as well. These compounds among others that contain hydroxyls are responsible for the radical scavenging effect [39,52]. The content of flavonoids (TFC) in the menthanol SP extract (F) was expressed as hyperoside percentage, and the TFC value was calculated as 0.29 ± 0.03%. Comparing with available literature data, this content is similar to that reported for *S. raeseri* and *S. scardica* [20,46]. It has been reported that light intensity, temperature and altitude could influence the biosynthesis of flavonoids [53]. 

Tannins are generally defined as naturally occurring polyphenolic compounds of high molecular weight. They have been accorded an important role in protecting plant tissues from herbivore attack [54]. Dietary supplementation of these compounds reduces the oxidative damage to cell membrane lipid, protein and nucleic acid due strong quenching property of free radicals [55]. Thus, these compounds also provide protection from cardiovascular, immune/autoimmune diseases and brain dysfunctions viz. Parkinson’s, Alzheimer’s, Huntington’s diseases [56]. The content of tannins, expressed as pyrogallol percentage, in the methanol SP extract (F) was presented as the mean value of three determinations, resulting as 0.22 ± 0.04%, which is quite lower than in the case of *S. scardica* [20].

### 2.4. Antimicrobial Activity

The SPEO antimicrobial potency, compared with that of each reference compound, is shown in Table 4. The EO showed high activity against methicillin-susceptible *Staphylococcus aureus* (MSSA) and methicillin-resistant *S. aureus* (MRSA), both at high (~5 × 10^5^ CFU/mL) and low (~5 × 10^3^ CFU/mL) inoculum. In fact, with respect to MSSA, the minimal inhibitory concentration (MIC) value was 0.307 mg/mL, with bactericidal activity obtained at 0.615 mg/mL; in the case of MRSA, even stronger activity was observed, with the MIC and minimal bactericidal concentration (MBC) values of 0.076 and 0.153 mg/mL, respectively.

In contrast, no effect was found for *E. coli*, carbapenem-susceptible and carbapenem-resistant *K. pneumoniae*, with the MIC values for both pathogens >2.46 mg/mL. Regarding the antifungal activity of SPEO, the MIC value was 2.46 mg/mL for *C. albicans*, without reaching fungicidal activity (MBC > 2.46 mg/mL). 

In addition to observed activity of SPEO against Gram(+) microorganisms and *C. albicans*, SP extracts have been investigated as well. With the aim to assess the antimicrobial efficacy, the extracts were analysed against MSSA, MRSA and *C. albicans*. The results are shown in Table 5. Similarly to the activity of SPEO, the highest efficacy was observed against both MSSA and MRSA strains, at high and low inoculum, for the extracts **C** and **F**, with bactericidal activity achieved at 3.09 mg/mL and 2.90 mg/mL, respectively. The extract **A** was bactericidal against MSSA and MRSA at high inoculum at the concentration of 4.74 mg/mL, whereas **D** had only a slight activity (i.e., 2 log10 CFU/mL reduction compared to the initial inoculum) against high inoculum of MRSA at 1.3 mg/mL, thus showing bacteriostatic effect. 

Furthermore, the potent fungicidal activity has been found for both **B** and **C** extracts, with the absence of growth at the concentration 0.75 mg/mL and 3.09 mg/mL, respectively. Our assumption was that the mentioned extracts, obtained at room temperature using non-polar extragens (*n*-hexane and 1,2-dichloroethane) might have resemblance of EO, taking into account that non-polar terpenoids, phenylpropanoids and, in some samples, diterpenoids are constituents of EOs. The extracts **A**, **D** and **F** lacked to show any effect against *C. albicans*. High resistance of each tested microorganisms has been shown in the case of the extracts **E**, **G** and **H**.

There are several reports on the antimicrobial activity of different *Sideritis* species. For instance, a strong inhibitory effect against *Staphylococcus epidermidis* was shown by *S. trojana* EO [57]. According to the authors, a higher content of oxygenated terpenes derivatives in this EO may be responsible for the better antimicrobial activity. Some Spanish *Sideritis* species showed great antimicrobial efficacy against Gram(+) bacteria and *C. albicans* fungal strain, with the lack of any activity against Gram(−) bacterial strains [58]. Similar results with a significant activity on Gram(+) bacteria were achieved with EOs of *S. curvidens* and *S. lanata* [59]. Six *Sideritis* taxa of Greek origin have been also analysed confirming this rule. *S. aureus* was found as the most sensitive strain, whereas *E. coli*, *K. pneumoniae* and *P. aeruginosa* were resistant to the tested oils [21]. *S. lanata* EO exhibited the best antimicrobial activity even comparable to the reference antibiotics in the case of *S. aureus* and *M. luteus*. Interestingly, one strain of *C. albicans* was more sensitive compared with the other that was resistant to all tested oils. The EOs of two *S. clandestine* subspecies exhibited good antifungal activity [21]. However, some authors reported the same activity on both Gram(+) and Gram(−) bacteria, as well as *C. albicans*, explaining it by the presence of α- and β-pinene as the main EO constituents [60]. Interestingly, the higher activity against Gram(−) strains has been reported for *S. italica*, particularly against *P. aeruginosa* [13].

Aside from EOs, *Sideritis* solvent extracts were also reported to possess significant antimicrobial activity. Considerable potency was reported for methanolic extracts of Turkish *S. ozturkii* and *S. caesarea* [61]. Good activity against *S. aureus* was shown for hexane extract of *S. scardica* [62]. Moreover, its EO, the ethanol extract and its different fractions, as well as the CO_2_-supercritical and conventional extracts have been analyzed on a series of bacterial and yeast strains [19]. Results indicated strong to moderate potency of all investigated extracts, with Gram(+) bacterial strains being more susceptible than the Gram(−) ones, with the exception of *Pasteurella multocida* and *Haemophilus* sp. The strongest antibacterial activity was reported against *Corynebacterium pseudotuberculosis* for the ethyl-acetate and *n*-butanol fractions, as well as for the EO. Interestingly, the mentioned solvent extracts exhibited moderate activity against MRSA with MIC values 0.64 mg/mL, while *E. coli* and *K. pneumoniae* proved to be the most resistant. According to the authors, the observed activity might be attributed to the presence of different terpenoids types, including diterpenes that have been reported to display important biological activities [19,63]. 

However, SP seems to be less investigated. To the best of our knowledge, there is only one report for the antimicrobial activity of its EO. In that study, the strong activity was found against *S. aureus* and *Micrococcus luteus* with MIC values of 0.006 mg/mL, whereas the good activity was reported against *S. epidermidis* and *C. albicans* (the MIC value of 0.024 mg/mL). That study also revealed the resistance of *Enterococcus faecalis*, *E. coli*, *P. aeruginosa* and *K. pneumoniae* strains [21]. As expected, due to similar EO chemical compositions, the results herein presented are mainly in agreement with those previously reported. The observed antimicrobial activities might be very interesting when considering their possible use as a part of the treatment of infections caused by MSSA, MRSA and *C. albicans*. The predominance of sesquiterpenoids (64.8%) could be addressed for the antimicrobial activity, particularly in the case of the opportunistic pathogen *S. aureus* strains. At least, this could be partly attributed to the (*E*)-caryophyllene content that has already demonstrated good activity against *S. aureus* [64,65,66]. Other main compounds bicyclogermacrene and germacrene D are known for their antimicrobial activity [67,68], whereas the spathulenol content could also have a synergy to the exhibited activity [69]. However, considering the phytocomplex, EO’s trace compounds could also be important as they could act synergistically and/or with antagonistic effects to the antimicrobial actions [64,70,71].

## 3. Materials and Methods

### 3.1. Plant Material

Aerial parts (*herba*) of wild growing SP were collected in June 2014 in a suburban area Šteke (42°40′94′′ N, 19°17′41.1′′ E) at an altitude of 134 m and about 5 km from Podgorica city (Montenegro) during the time of flowering. Air-drying of the collected plant was performed in a shady place for approximately 20 days, packed in paper bags and kept in a dark and cool place. A voucher specimen (No. DB-SP/01) has been deposited at the University of Montenegro, Department of Biology. 

Taxonomic identification was performed by M.B. (the author) and was conducted according to the official European flora [22].

### 3.2. EO Extraction

Fifty grams of semi-crushed air-dried plant material and 800 mL of distilled water were subjected to hydrodistillation for 3 h using a Clevenger-type apparatus to produce EO. The accumulated oil/water double phase was extracted 3 times with 20 mL of diethyl ether. The organic layers were dried over anhydrous sodium sulfate (Na_2_SO_4_), filtered and deprived of the solvent in vacuo to furnish a yellow pale oil. Prepared SP oil (SPEO) was stored in a tightly closed dark vial until further analysis.

### 3.3. Solvent Extraction

Two independent ways of extraction were applied. Firstly, the finely ground sample of SP herba (15 g) was macerated with methanol (300 mL) for 48 h. For maceration extraction, the material was placed in an Erlenmeyer flask, the corresponding amount of solvent was added with the sample left to macerate in the dark on a shaker at room temperature. The obtained extract was filtered, and half of the crude methanol extract (**A**) was partitioned into 4 parts, concentrated in vacuum evaporator, re-dissolved in distilled water, shaken vigorously and additionally extracted with 4 solvents. Using a separating funnel, 35 mL of each crude extract part (**A**) was extracted 3 times with 20 mL of each solvent: *n*-hexane, 1,2-dichloroethane, ethyl-acetate and chloroform, resulting in **B**, **C**, **D** and **E** extract fractions, respectively. The extracts were dried under reduced pressure and used to determine the antimicrobial activity.

In addition, shape-dried powdered SP samples were extracted in Soxhlet apparatus for 24 h according to the standard procedure. Ten grams of each sample was separately extracted with 300 mL of 3 different solvents. Methanol, acetone and diethyl-ether were used, and obtained extracts were labelled as **F**, **G** and **H**, respectively. After the mixtures were evaporated in vacuum, the final extracts were stored in glass vials in refrigerator until further use.

### 3.4. EO Chemical Analysis

Gas chromatography (GC) analysis was carried out on a HP-5890 Series II GC apparatus (Hewlett-Packard, Waldbronn, Germany), equipped with a split-splitless injector and automatic liquid sampler, attached to a HP-5 column (25 m × 0.32 mm, film thickness 0.52 μm) and fitted with an flame ionization detector (FID). Carrier gas flow rate (H_2_) was 1 mL/min, split ratio 1:30, injector temperature was 250 °C, detector temperature 300 °C, while column temperature was linearly programmed from 40 to 260 °C (at rate of 4 °C/min), and then kept isothermally at 260 °C for 10 min. Solutions of samples (23.9 mg/mL dichloromethane) were consecutively injected in amount of 1 μL. Area percentage reports, obtained as result of standard processing of chromatograms, were used as a base for the quantification analysis.

The same analytical conditions as those mentioned for GC-FID were employed for GC-MS analysis, along with a column HP-5MS (30 m × 0.25 mm, film thickness 0.25 μm), using a HP G 1800C Series II GCD system (Hewlett-Packard, Palo Alto, CA, USA). Helium was used as carrier gas. The transfer line was heated at 260 °C; mass spectra were acquired in electron ionization (EI) mode (70 eV); in the 40–450 *m/z* range. An amount of 0.2 μL of sample solution in dichloromethane (23.9 mg/mL) was injected. The components of the EOs were identified by comparison of their mass spectra to those from the Wiley 275 and National Institute of Standards and Technology/National Bureau of Standards (NIST/NBS) libraries, using different search engines. Identification of the compounds was achieved by comparing their retention indices and mass spectra with those found in the iterature [72] and supplemented by the Automated Mass Spectral Deconvolution and Identification System software (AMDIS ver. 2.1, Manufacturer, National Institute of Standards and Technology (NIST), Standard Reference Data Program, Gaithersburg, MD, USA), GC-MS Libraries. The experimental values for retention indices were determined by the use of calibrated Automated Mass Spectral Deconvolution and Identification System Software (AMDIS ver. 2.1, National Institute of Standards and Technology (NIST), Standard Reference Data Program, Gaithersburg, MD, USA), GC-MS Libraries, compared to those from available literature [72] and used as an additional tool to confirm the MS findings. The relative proportion of the EO constituents was expressed as percentages obtained by peak area normalization, all relative response factors being taking as one.

### 3.5. Determination of Total Phenols Content (TPC)

TPC was determined by the Folin–Ciocalteu method. In addition, 100 μL of methanolic solution of an SP dry extract (F) (15.63, 7.81 and 3.96 μg/mL final quantity) was mixed with 0.75 ml of Folin–Ciocalteu reagent (previously diluted 10-fold with distilled water) and allowed to stand at 22 °C for 5 min; 0.75 mL of sodium bicarbonate (60 g/L) solution was added to mixture. After 90 min at 22 °C, the absorbance was measured at λ_max_ 725 nm. Gallic acid (0–100 mg/L) was used for calibration of a standard curve. The calibration curve showed the linear regression at r > 0.99, and the results were expressed as milligrams of gallic acid equivalents per gram of plant extract dry weight (mg GAE/g DW). Triplicate measurements were taken and data were presented as mean ± standard deviation (SD) [73].

### 3.6. Determination of Total Tannins Content (TTC)

TTC was calculated using the method described in the European Pharmacopoeia, Ph. Eur. 6.0 [74]. Shortly after, decoctions prepared from the investigated extract F were treated with phosphomolybdotungstic reagent in alkaline medium after and without treatment with hide powder. The absorbance was measured by UV-VIS Spectrophotometer HP 8453 (Agilent Technologies, Santa Clara, CA, USA) at λ_max_ 760 nm. From the difference in absorbance of total polyphenols and polyphenols not adsorbed by hide powder (*A*_1_ and *A*_2_, respectively), the content of tannins expressed as pyrogallol (%, *w*/*w*) was calculated from the expression:

(1) $$TTC=\frac{62.5\times \left(A_{1}-A_{2}\right)\times m_{2}}{A_{3}\times m_{1}}$$

where *m*_1_ represented mass of the sample to be examined (in grams), and *m*_2_ was mass of pyrogallol (in grams), *A*_3_ represented the absorbance of pyrogallol standard solution. The results represented the mean ± SD of three determinations.

### 3.7. Determination of Total Flavonoid Content (TFC)

TFC expressed as hyperoside was calculated using the method described in the Deutsches Arzneibuch, DAB 10. Briefly, the plant extract (F) was extracted with acetone/HCl under reflux condenser; the AlCl_3_ complex of the flavonoid fraction extracted by ethyl acetate was measured by UV-Vis Spectrophotometer HP 8453 at λ_max_ 425 nm. The content of flavonoid, expressed as hyperoside percentage, presented the mean ± standard deviation of three determinations.

### 3.8. Bacterial Strains and Growth Media

For the antimicrobial activity determination, the following microorganisms have been used: methicillin-susceptible *Staphylococcus aureus* (MSSA (ATCC 29213)) and methicillin-resistant *S. aureus* (MRSA (clinical strain)), *Escherichia coli* (ATCC 25922), carbapenem-susceptible *Klebsiella pneumoniae* (clinical strain), carbapenem-resistant *K. pneumoniae* (clinical strain) and *Candida albicans* (ATCC 14053).

After bacterial storage on cryovial bead preservation system (Microbank; Pro-Lab Diagnostics, Richmond Hill, Ontario, ON, Canada) at −80 °C, inoculum was prepared by spreading one cryovial bead on blood agar plate and incubated overnight at 37 °C. One colony was re-suspended in 5 mL tryptic soy broth (TSB) and incubated at 37 °C without shaking. Overnight cultures were then adjusted to a turbidity of 0.5 McFarland, corresponding to ≈ 1 × 10^8^ CFU/mL. 

### 3.9. Antimicrobials Agents and EO/Extracts Solutions Preparation

Antimicrobial agents used as reference were provided as purified powder by the manufacturer (Sigma Aldrich, Milan, Italy). Stock solutions at different concentrations were prepared in sterile and pyrogen-free 0.9% saline or water, according to the manufacturer’s instructions.

SPEO and SP extracts were dissolved in 50% dimethylsulphoxide (DMSO) or water, as appropriate, in order to obtain complete solubilization. Used concentration of DMSO did not interfere with bacterial and fungal viability.

### 3.10. Antimicrobial Assay

Given that the starting solutions were limpid, both minimal inhibitory and bactericidal concentrations (MICs/MBCs) of each reference compound and EO were determined. In contrast, the starting solution of SP extracts was opalescent; thus, only the MBCs were evaluated.

Two-fold serial dilutions of each antimicrobial agent, SPEO and SP extracts were prepared in 2 mL Mueller Hinton broth (MHB) in borosilicate glass tubes and incubated for 24 h at 37 °C. The MIC value was defined as the lowest concentration of antibiotic that completely inhibited visible growth whereas bactericidal activity was defined as ≥3-log10 CFU/mL reduction of the initial bacterial count after 24 h of incubation [75]. Cation-adjusted Mueller Hinton (CAMHB) and Sabouraud broth were used for bacteria and fungi, respectively.

The following antimicrobial agents were used as references: vancomycin (VAN, Sigma Aldrich, purity >99%) and rifampin (RIF, Sigma Aldrich, purity ≥97%) for Gram-positive microorganisms, meropenem (MEM, Sigma Aldrich, purity ≥98%) for Gram-negative bacteria, and fluconazole (FLU, Sigma Aldrich, purity ≥98%) for fungi.

The used bacterial inoculum was ~5 × 10^3^ CFU/mL. Given the high effect observed against MSSA and MRSA, we tested the EO and SP extracts even at higher inoculum (~5 × 10^5^ CFU/mL), which represents the likely bacterial amount in case of systemic infections [76].

All the experiments were performed in duplicate.

## 4. Conclusions

Plants have provided the source of inspiration for novel drug compounds. Their extracts and EOs have been shown as useful alternative antimicrobial substances, and numerous studies are continuously published on their uses and applications. In this context, investigations on SP are rather rare. Thus, to initially fulfill this gap in this work, we report for the first time a comprehensive study on EO’s chemical composition and its related antimicrobial activity, as well as of different solvent extracts from SP material collected in Montenegro.

SPEO could be described as sesquiterpenoids-rich one (64.8%), with only 10.1% of oxygenated mono- and diterpene fractions. Among the 43 recognized chemical components bicyclogermacrene, germacrene D, (*E*)-caryophyllene and spathulenol were the most abundant. The strongest antimicrobial activities were found against MSSA and especially MRSA strains, as well as against *C. albicans* strain. These findings were confirmed by several solvent extracts corroborating this plant’s usage as a part of the treatment for infectious diseases caused by these opportunistic pathogens. Undoubtedly, further studies are required to evaluate the contribution of SPEO’s compounds in the antimicrobial activity expression. The synergism, antagonism and additive effects of the EO’s components require additional investigation to elucidate the mechanisms underlying their biological activity, for the purpose of accessing new natural antiseptics applicable in the pharmaceutical and food industry. Additionally, studies on SP extracts can be proposed. 

Although present in SP, different phenolic compounds, flavonoids and tannins obviously had not shown the same antimicrobial activity as its EO. Generally, derivatives of 2-phenylchromen-4-one (bioflavonoids) and 2-phenyl-3,4-dihydro-2*H*-chromen-3-ol (tannins), as subgroups within the larger flavonoid group, are considered to be the main groups of active constituents, and, in many national and international pharmacopoeias, these groups of compounds are used for their standardization and quality control. Thus, besides the determination of EO contents, its chemistry profile and antimicrobial potential, we performed the characterization of the plant material in terms of other secondary metabolites.

Bearing in mind the numerous findings on *Sideritis* species, it can be concluded that SP provides a wide range of application in different fields such as phytochemistry, pharmacology, toxicology or pharmacognosy.

## Figures and Tables

**Table 1:** Maceration extraction: final extracts yield (in grams).

Sample Name	Solvent	Yield (Grams)
A	methanol	0.76
B	*n*-hexane	0.03
C	1,2-dichloroethane	0.03
D	ethyl-acetate	0.05
E	chloroform	0.02

**Table 2:** Soxhlet extraction: final extracts yield %.

Sample Name	Solvent	Yield % ^1^
F	methanol	0.254
G	acetone	0.176
H	diethyl-ether	0.191

^1^ calculated per plant material weight.

**Table 3:** Chemical composition of SPEO. Values are in weight %.

# ^1^	Compound Name	KI ^2^	%	# ^1^	Compound Name	KI ^2^	%
1	1,8-cineole	1026	0.3	*23*	viridiflorol	1592	0.8
2	camphor	1141	0.3	*24*	8-cedren-13-ol	1688	1.1
3	*trans*-chrysanthenyl acetate	1235	0.2	*25*	myristic acid	1770	1.1
4	α-terpinyl acetate	1346	3.1	*26*	isopropyl tetradecanoate	1828	1.7
5	γ-ylangene	1374	0.2	*27*	hexahydrofarnesyl acetone	1848	6.9
6	α-copaene	1377	1	*28*	(5*E*,9*E*)-farnesyl acetone	1913	0.5
7	β-bourbonene	1387	0.4	*29*	phytol	1942	0.9
8	β-(*E*)-demascenone	1383	0.5	*30*	palmitoleic acid	1953	0.5
9	β-elemene	1384	0.6	*31*	palmitic acid	1959	7.6
10	(*E* or β)-caryophyllene	1417	7.9	*32*	isopropyl hexadecanoate	2024	0.6
11	*endo*-arbazol	1433	0.2	*33*	manool	2056	0.9
12	aromadendrene	1439	0.3	*34*	*epi*-manool	2059	0.3
13	α-humulene	1452	0.4	*35*	octadecanol	2077	0.6
14	β-farnesene	1454	0.4	*36*	phytol acetate	2218	1.9
15	γ-muurolene	1478	0.9	*37*	3α-hydroxy manool	2297	0.9
16	germacrene D	1484	8	*38*	3-deoxy estradiol	2300	0.8
17	bicyclogermacrene	1500	23.8	*39*	torulosol	2360	0.8
18	10-epi-italicene ether	1515	1.6	*40*	pentacosane	2500	0.7
19	δ-cadinene	1522	1	*41*	hexacosane	2600	0.5
20	spathulenol	1577	5.5	*42*	heptacosane	2700	0.1
21	caryophyllene oxide	1582	2	*43*	nonacosane	2900	1.1
22	globulol	1590	1.5		Unidentified compounds		9.6

^1^ # indicates the compound identification number; ^2^ Kovats index, experimentally determined.

**Table 4:** Activity of SPEO against different bacterial and fungal strains detected through microbroth dilution.

Microorganism	MIC Low Inoculum ° (mg/mL)	MBC Low Inoculum ° (mg/mL)	MIC High Inoculum ^§^ (mg/mL)	MBC High Inoculum ^§^ (mg/mL)	Activity	MIC Reference Antimicrobial (mg/L)
**MSSA** **(ATCC 29213)**	0.307	0.615	0.307	0.615	Bactericidal	0.5 (VAN)–0.03 (RIF)
MRSA *	0.076	0.153	0.076	0.153	Bactericidal	1 (VAN)–0.06 (RIF)
***E. coli*** **(ATCC 25922)**	>2.46	>2.46	-	-	No effect	0.125 (MEM)
**CS** *K. pneumoniae* *	>2.46	>2.46	-	-	No effect	0.25 (MEM)
**CR** *K. pneumoniae* *	>2.46	>2.46	-	-	No effect	16 (MEM)
***C. albicans*** **(ATCC 14053)**	2.46	>2.46	-	-	Fungistatic	0.25 (FLU)

° Low inoculum: 5 × 10^3^ CFU/mL; ^§^ High inoculum: 5 × 10^5^ CFU/mL; * clinical strain; MSSA: methicillin-susceptible *S. aureus*; MRSA: methicillin-resistant *S. aureus*; CS: carbapenem-susceptible; CR: carpabenem-resistant; ATCC: American Type Culture Collection; MIC: Minimal Inhibitory Concentration Value; MBC: Minimal Bactericidal Concentration Value; CS: carbapenem-susceptible; CR: carbapenem-resistant; VAN: vancomycin; RIF: rifampin; MEM: meropenem; FLU: fluconazole.

**Table 5:** Activity of different *Sideritis romana* L. subsp. *purpurea* (Tal. ex Benth.) Heywood extracts against methicillin-susceptible *S. aureus*, methicillin-resistant *S. aureus* and *C. albicans.* Low and high inoculum are 5 × 10^3^ and 5 × 10^5^ colony forming units (CFU)/mL, respectively.

Sample Name	MBC MSSA (ATCC 29213) Low Inoculum (mg/mL)	MBC MSSA (ATCC 29213) High Inoculum (mg/mL)	MBC MRSA ° Low Inoculum (mg/mL)	MBC MRSA ° High Inoculum (mg/mL)	MBC *C. albicans* (ATCC 14053) Low Inoculum (mg/mL)	Antibacterial Activity	Antifungal Activity
**A**	18.99	4.74	4.74	4.74	>18.99	Bactericidal	No effect
**B**	>0.75	>0.75	>0.75	>0.75	0.75	No effect	Fungicidal
**C**	3.09	3.09	3.09	3.09	3.09	Bactericidal	Fungicidal
**D**	>1.30 *	>1.30	>1.30	>1.30 **	>1.30	Bacteriostatic	No effect
**E ^#^**	>0.89	>0.89	>0.89	>0.89	>0.89	No effect	No effect
**F**	2.90	2.90	2.90	2.90	>5.80	Bactericidal	No effect
**G**	>5.25	>5.25	>5.25	>5.25	>5.25	No effect	No effect
**H**	>3.07	>3.07	>3.07	>3.07	>3.07	No effect	No effect

° clinical strain; * 1log10 CFU/mL reduction compared to the initial inoculum was observed; ** 2log10 CFU/mL reduction compared to the initial inoculum was observed; MSSA: methicillin-susceptible *S. aureus*; MRSA: methicillin-resistant *S. aureus;* MBC: Minimal Bactericidal Concentration; ^#^ solubilisation of this extract was not optimal.
